# Ranolazine promotes muscle differentiation and reduces oxidative stress in C2C12 skeletal muscle cells

**DOI:** 10.1007/s12020-016-1181-5

**Published:** 2016-12-08

**Authors:** Terruzzi Ileana, Montesano Anna, Senesi Pamela, Vacante Fernanda, Benedini Stefano, Luzi Livio

**Affiliations:** 1 Diabetes Research Institute, Metabolism, Nutrigenomics and Cellular Differentiation Unit, San Raffaele Scientific Institute, 60 Olgettina street, 20132 Milan, Italy; 2 Department of Biomedical Sciences for Health, University of Milan, Milan, Italy; 3 Metabolism Research Center, IRCCS Policlinico San Donato, San Donato Milanese, Milan, Italy

**Keywords:** Ranolazine, Muscle differentiation, Oxidative stress

## Abstract

**Purpose:**

The purpose of this study is to investigate Ranolazine action on skeletal muscle differentiation and mitochondrial oxidative phenomena. Ranolazine, an antianginal drug, which acts blocking the late INaL current, was shown to lower hemoglobin A1c in patients with diabetes. In the present study, we hypothesized an action of Ranolazine on skeletal muscle cells regeneration and oxidative process, leading to a reduction of insulin resistance.

**Methods:**

10 μM Ranolazine was added to C2C12 murine myoblastic cells during proliferation, differentiation and newly formed myotubes.

**Results:**

Ranolazine promoted the development of a specific myogenic phenotype: increasing the expression of myogenic regulator factors and inhibiting cell cycle progression factor (p21). Ranolazine stimulated calcium signaling (calmodulin-dependent kinases) and reduced reactive oxygen species levels. Furthermore, Ranolazine maintained mitochondrial homeostasis. During the differentiation phase, Ranolazine promoted myotubes formation. Ranolazine did not modify kinases involved in skeletal muscle differentiation and glucose uptake (extracellular signal-regulated kinases 1/2 and AKT pathways), but activated calcium signaling pathways. During proliferation, Ranolazine did not modify the number of mitochondria while decreasing osteopontin protein levels. Lastly, neo-formed myotubes treated with Ranolazine showed typical hypertrophic phenotype.

**Conclusion:**

In conclusion, our results indicate that Ranolazine stimulates myogenesis and reduces a pro-oxidant inflammation/oxidative condition, activating a calcium signaling pathway. These newly described mechanisms may partially explain the glucose lowering effect of the drug.

## Introduction

Ranolazine (RAN), a selective inhibitor of the late sodium current (INaL), has proven effective in the treatment of chronic angina.

A clinical trial, denominated TERISA, was recently performed to examine the association between different classes of glucose-lowering medications and angina frequency. TERISA was a randomized, double-blind, placebo-controlled trial across 104 sites in 14 countries in which patients with type II diabetes, documented coronary artery disease, and a 3-month history of stable angina were randomized to twice daily doses of RAN or a placebo for 8 weeks. The TERISA clinical trial has, interestingly, detected a RAN beneficial effect in reducing glycosylated hemoglobin and in the occurrence of fasting glucose in patients without previous evidence of diabetes [1]. Ning et al. [2] showed that RAN-treated mice had increased β-cell mass with a lower degree of apoptosis leading to a higher glucose-stimulated insulin secretion. More recently, Rizzetto et al. demonstrated that RAN stimulates insulin secretion increasing calcium influx in human islets and in rat INS-1E cells [3]. Previous data suggest that RAN may be a novel antidiabetic agent acting on the β-cell function. Insofar no evidence is available that RAN may also act promoting peripheral insulin sensitivity [4]. Skeletal muscle insulin resistance (IR) is characterized by a reduced insulin stimulation of signaling pathways [5] (AKT/p70S6) leading to translocation of glucose transporter-4 (GLUT4) toward the plasma membrane [6]. An insulin-independent mechanism also exists in addition to the insulin-mediated translocation of GLUT4; the former being mediated by the rise of intracellular calcium [7], which activates calmodulin-dependent kinases (CaMKII).

Calcium signals are associated to increased levels of reactive oxygen species (ROS) causing oxidative stress and initiating the progress of insulin resistance [8]. ROS overproduction is involved in a variety of myopathies including diabetic myopathy [9]. Osteopontin (OPN), a factor of skeletal muscle inflammatory process [10], is overexpressed in IR muscles. An acute increase of OPN expression is critical for tissue remodeling taking place following cellular stress or injury, but its chronic overexpression results in chronic inflammation and muscle fibrosis [11].

Myoblasts differentiation is a complex process, involving a sequential cascade of regulatory events [12] concerning muscle-specific transcription factors. Myogenic regulatory factors (MRFs) Myf5 and MyoD are expressed in the proliferating myogenic cells (myoblasts) that withdraw from the cell cycle and start differentiation. Postmitotic myocytes, expressing Myosin Heavy Chain (MyHC) and Myogenin, elongate and fuse to repair existing damaged myofibers or to form new multinucleated myofibers [13]. Myocyte differentiation involves complex interactions between transcription factors and signal transduction pathways. The extracellular signal-regulated kinase 1/2 (ERK) and AKT/p70S6 signaling pathways are the most intensively studied mechanisms regulating both proliferation and differentiation [14, 15]. These pathways cross communicate extensively and fine tune reciprocally [16]. Moreover, there is a widespread interaction between CaMKs and ERK1/2 and AKT signaling pathways [17].

It is presently unknown whether RAN-induced INaL modification may influence proliferation, differentiation and hypertrophy of skeletal muscle cells. In this paper we address the hypothesis that RAN plays a role in muscle development and metabolism utilizing an insulin-dependent mechanism.

We demonstrate an insulin-dependent anabolic effect of RAN in modulating skeletal muscle myogenic process. Our results could constitute a proof of principle for the future development of therapeutic strategies to ameliorate insulin resistance improving muscle formation, calcium signaling, oxidative stress and cell regeneration.

## Material and methods

### Materials

C2C12 mouse cells were purchased from the European Collection of Animal Cell Cultures (ECACC), reagents from Sigma Chemical Co. (St. Louis-MO, USA).

Primary antibodies against: Calnexin (H-70), GAPDH (FL-335), AKT (C-20), CaMKII (M-176), pCaMKIIalpha (Thr286), MyHC (H-300), Myf5 (C-20), MyoD (C-20), Myogenin (D-10), p53 (FL-393), p70S6 (C-18), pp70S6 (C-18), ERK1 (K-23), ERK2 (C-14), pERK1/2 (E-4), p21 (C-19), OPN (K-20), peroxidase-conjugated secondary antibodies for Western blot analysis and Rhodamine-conjugated antibodies for Immunofluorescence studies were purchased from Santa Cruz Biotechnology (Santa Cruz-CA, USA). Primary antibody Phospho-AKT (Ser473-D9E-XP^TM^) was purchased from Cell Signaling Technology (Danvers-MA, USA).

Fluorescently-labeled phalloidin (AlexaFluor^®^488-Invitrogen) was purchased from Life Technologies (Carlsbad-CA, USA).

CytoPainter Mitochondrial Staining Kit-Green (AB 112143) was purchased from Prodotti Gianni (Milano-Italy), Cell ROX^®^ Oxidative Stress Reagents Kit (C10443) from Thermo Fisher Scientific, Life Technologies Italia (Monza-Italy).

### Cell culture

C2C12 cells were maintained at 37 °C in 5 % CO_2_ humidified atmosphere in a growth medium (GM) containing Dulbecco modified Eagle medium (DMEM) with 20 % (v/v) fetal bovine serum, 1 % penicillin streptomycin and 1 % l-glutamine. 70 % confluent cells were placed in differentiation medium (DM) (DMEM with 1 % horse serum-HS, antibiotics and 1 % l-glutamine). Myoblast C2C12 immortalized cells spontaneously fuse and differentiate into multinucleated myotubes as a result of either the achievement of myoblast confluence (GM) or the removal of serum growth factors (DM). In our in vitro differentiation model, early myotubes appeared 24–48 hours (h) after serum starvation and neo myotubes formation was completed in 72 h [18].

### C2C12 myoblasts growth curve and viability test

**Fig. 1:**
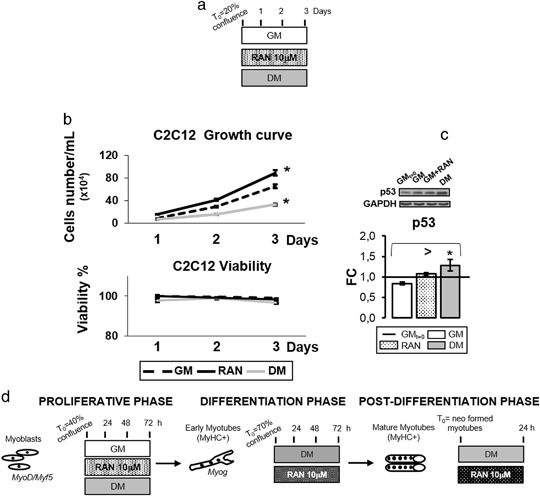
RAN effect on cell growth. **a** Experimental scheme for growth curve and viability determination. **b** C2C12 cells were seeded in 60 × 15 mm culture dishes at 20 % confluence and grown in GM medium with or without RAN, and DM medium. Medium was changed every 24 h and the experiment lasted until control cells achieved 40 % of confluence (3 days). As shown in panel **b**, RAN increases C2C12 proliferative potential without cytotoxic effects. **c** To confirm the results obtained by growth curve, we analyzed p53 protein content: Western blot analysis (panel **c**) shows the p53 significant increment in DM condition, confirming an inhibition of the cell cycle only in this condition, as reported in literature. In GM and GM-RAN cells p53 levels remain comparable with basal condition. **d** Experimental scheme for RAN treatment during proliferative, differentiation and post differentiation phases. Representative immunoblots of analyzed proteins are shown. Anova test: >*p *≤ 0.05, *t* test: * *p* ≤ 0.05 vs. GM

C2C12 myoblasts growth curve was performed as described [19]. Preliminary dose-response assay established 10 μM the effective dose for the in vitro treatment (data not shown). Briefly, C2C12 cells were plated in 60 × 15 mm culture dishes in GM with or without RAN and DM until achievement of 40 % confluence (Fig. 1a). The cells were stained with trypan blue and counted using a hemocytometer on a daily basis.

The average values for each single day were used to plot a growth curve.

Cell viability was calculated by dividing the non-stained viable cell count by the total cell count. Morphological changes were examined on a daily basis.

### Experimental procedures

Cells in the proliferative phase (from 40 to 70 % confluence), differentiating myocytes and neo myotubes were treated with 10 µM RAN. RAN was not added in the medium in the control cells (Fig. 1d).

### Western blot analysis

Cell protein extracts were obtained by using RIPA buffer [18]. Aliquots of 30 µg supernatant proteins were resolved on SDS-PAGE gel and transferred onto nitrocellulose membrane, incubated with specific primary antibodies and subsequently with HRP conjugated anti-species-specific secondary antibodies. Antibodies anti calnexin or GAPDH were used to confirm equal protein loading per sample. Quantitative measurement of immune-reactive bands intensities, detected with an enhanced chemiluminescence method (Amersham Pharmacia Biotech, Piscataway-NJ, USA), was performed by densitometric analysis using Scion Image software (Scion Corporation, Frederick-MD, USA). Data were converted into fold-changes (FC) of the control cells, as described [19].

### Immunofluorescence

C2C12 cells were fixed and permeabilized as described [18], were blocked with PBS containing 1 % bovine serum albumin. Slides or cells were then immuno-stained with specific Rhodamine- or FITC-conjugated antibodies and nuclei were revealed with DAPI staining.

The MITO CytoPainter mitochondrial indicator is a hydrophobic compound that easily permeates intact live cells and becomes trapped in mitochondria after it enters the cells. This staining was performed on C2C12 live cells during the proliferative phase and all the differentiation phases.

Cell ROX^®^ Oxidative Stress Reagents are fluorogenic probes designed to reliably measure ROS in live cells. The cell-permeable reagents are non-fluorescent or very faintly fluorescent while in a reduced state, and during oxidation exhibit a strong fluorogenic signal. Cell ROX^®^ Orange Reagents are localized in the cytoplasm. This staining was performed on C2C12 live cells during the proliferative phase.

Slides were mounted with Moviol. Cells were observed using Nikon Eclipse 50I microscopy and images were captured using Nis-Elements D 4.00 software (Nikon Instruments Europe BV-Netherlands).

Data were displayed and analyzed using Adobe^®^ Photoshop^®^CS4.

### Immunofluorescence quantification

Automated quantification on the immunofluorescence signal was performed using Image J program (http://imagej.nih.gov/ij/). On Image J, myotubes captured in phase contrast images were manually (Freehand selection function) measured. For each treatment condition we quantified myotubes from many different images. Measurements made here, were manually recorded or exported as a .txt file for further analysis in Microsoft Excel to complete statistical analysis.

## Statistical analysis

All experiments were performed three times. Data are presented as the mean ± SD. Statistical significances were assessed by *t*-test or ANOVA tests as appropriate. Results were considered significant when *p* ≤ 0.05.

## Results

### RAN effect on cell growth

Cell cycle arrest and acquisition of skeletal muscle-specific phenotype represent two important features of the myogenic differentiation program [15, 20]. C2C12 were cultured in a growth media with or without 10 µΜ RAN for 1, 2, 3 days (Fig. 1a) to examine RAN action on cell growth. With respect to GM (Fig. 1b), RAN increases C2C12 proliferation while DM arrests it. Viability assay graph assessed the absence of cell mortality in all conditions (Fig. 1b). Figure 1c confirms that the cell cycle inhibitor p53 content is not influenced by RAN while is significantly higher in DM.

### RAN action on proliferative myoblasts

**Fig. 2:**
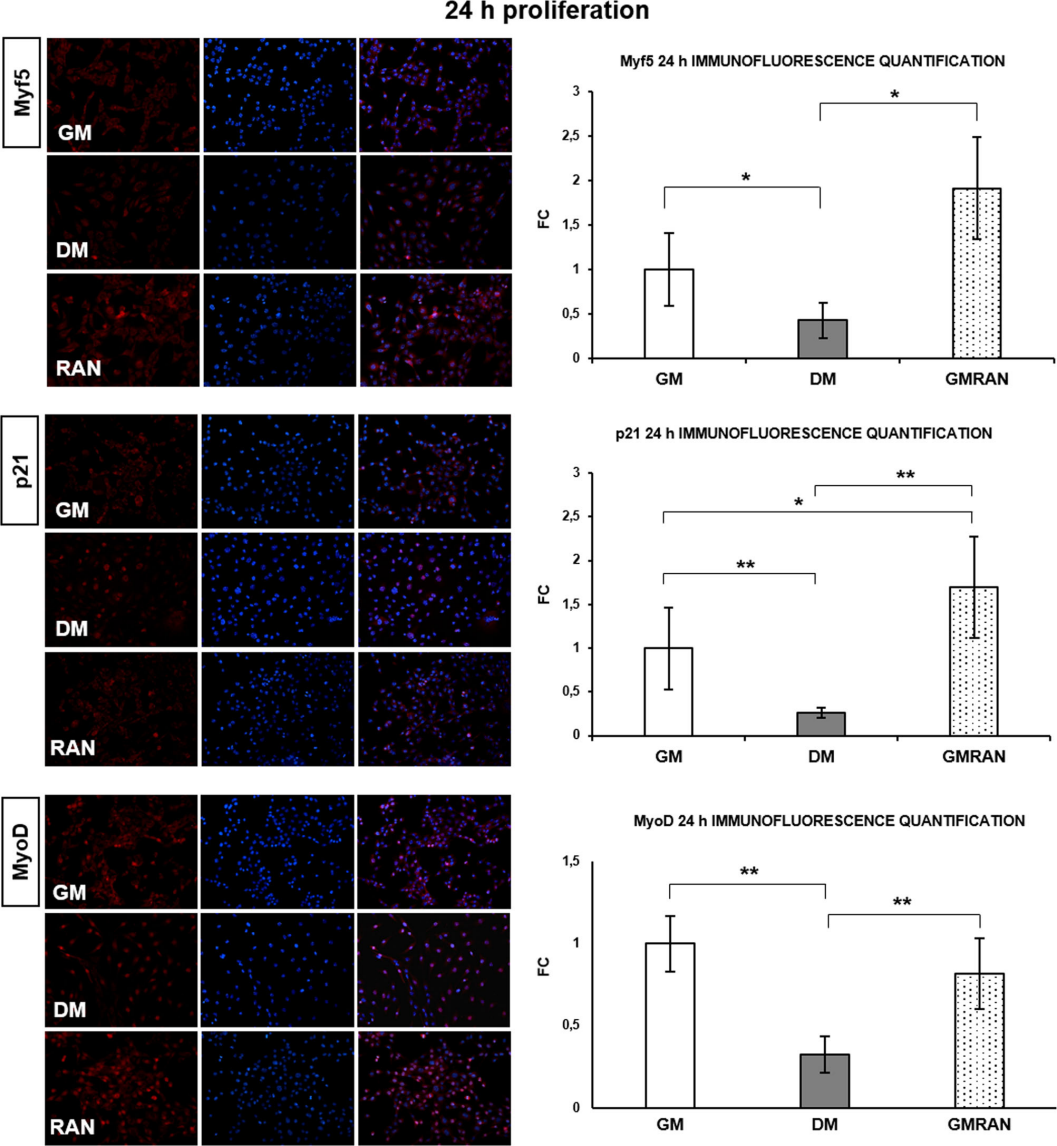
RAN action on proliferative myoblasts: 24 h. To investigate whether RAN could enhance myogenic phenotype acquisition, we analyzed myoblasts morphology and Myf5, p21 and MyoD protein expression. Immunofluorescence images and quantification (20X): RAN increased the signal of Myf5, p21 and MyoD proteins in respect of GM and DM at 24 h. These images showed the important morphological changes and the different localization of the signal in myoblasts treated with RAN with respect to GM and DM control. Objective: 20X. *t* test: *p ≤ 0.05 or **0.01 vs. GM

We studied the synthesis of p21 and MRFs [20, 21] to investigate RAN ability to promote myogenic phenotype acquisition. Figure 2 reveals how RAN treated cells at 24 h significantly increased expression of Myf5, MyoD and p21 compared with proliferative control (GM) and appear to have an intracellular localization of protein signal and morphological features comparable to differentiation control (DM).

**Fig. 3:**
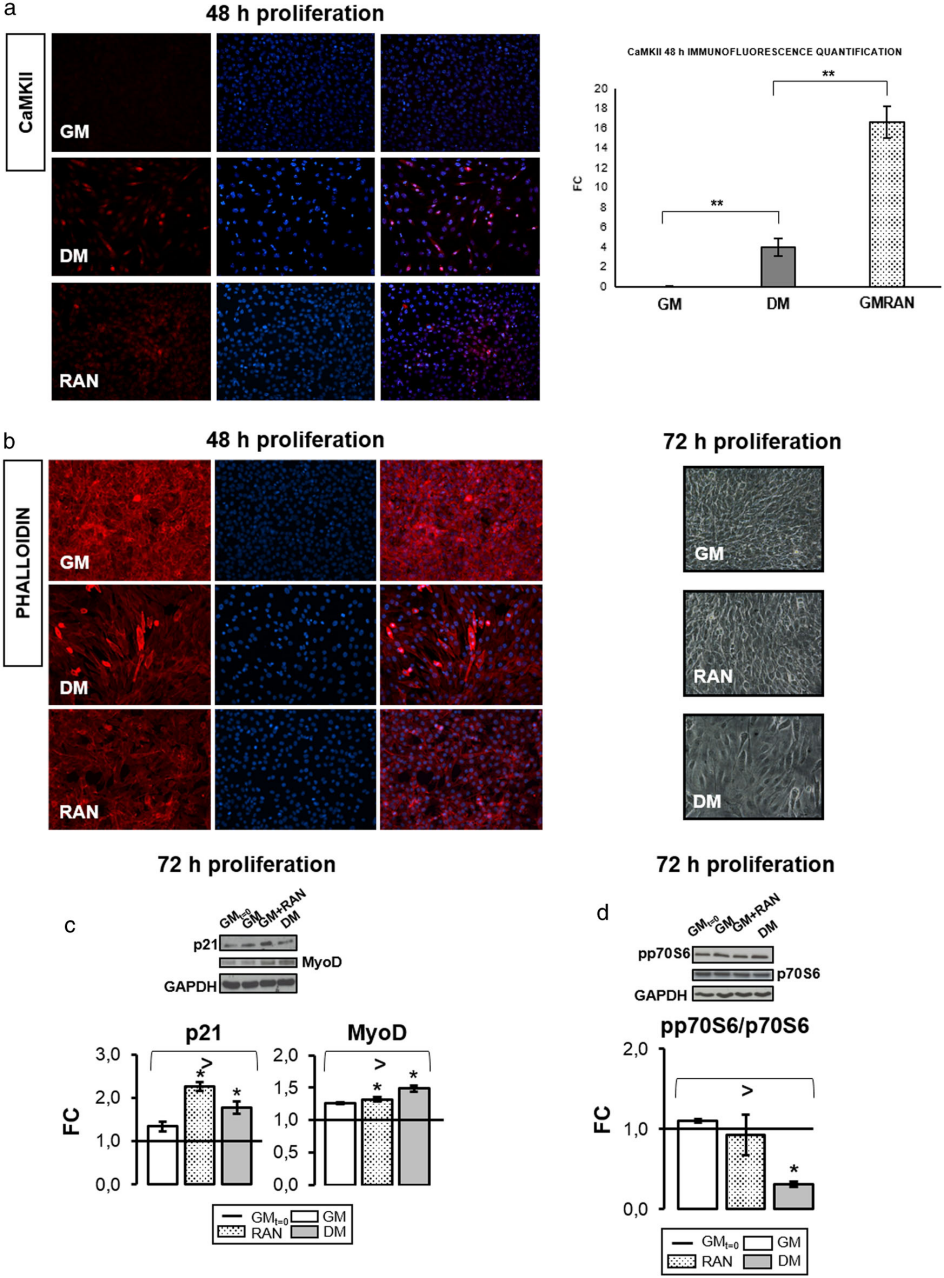
RAN action on proliferative myoblasts: 48–72 h. Intracellular calcium signaling and p70S6 kinase play an important role in the early stages of myogenesis. We studied whether RAN could act on CaMKII protein expression and p70S6 activation during proliferation phase. **a** Immunofluorescence analysis and quantification (20X): RAN increased the signal of CaMKII protein expression compared with GM at 48 h. **b** Phalloidin staining and phase contrast images corroborated the morphological changes observed in proliferative myoblasts (Fig. 2). **c** Western blot analysis: RAN significantly increased MyoD and p21 protein content at 72 h of proliferation. **d** Western blot analysis: RAN did not induce myoblast proliferation activating p70S6 kinase at 72 h. Representative immunoblots are shown. Objective: 20X. Anova test: >*p *≤ 0.05, *t* test: *p ≤ 0.05 or **0.01 vs. GM

Since intracellular calcium signaling is involved in the early stages of myogenesis [17,22], we tested whether RAN modulates CaMKII protein expression at 48 h of proliferation. RAN treatment not only induces a significant increase of CaMKII protein synthesis in C2C12 cells compared with GM control (Fig. 3a), but it also appears to induce cells to lose their circular shape, typical of the active proliferation state, to take an elongated morphology, superimposable to DM control morphology. Figure 3b well describes this evidence elucidating RAN role in the transition from undifferentiated myoblasts to myocytes. To further confirm our assumption, Western blot assay in Fig. 3c showed a significant increment of MyoD and p21 protein content in RAN-treated cells and DM compared with GM, suggesting a RAN stimulus to differentiation similar to DM. We also investigated if RAN regulates the p70S6 kinase activation. In fact, p70S6 kinase plays a role in protein synthesis and in cell growth control during the G1 phase [15]. Figure 3d shows that RAN did not induce p70S6 kinase activation.

### RAN action on C2C12 myoblasts mitochondria

**Fig. 4:**
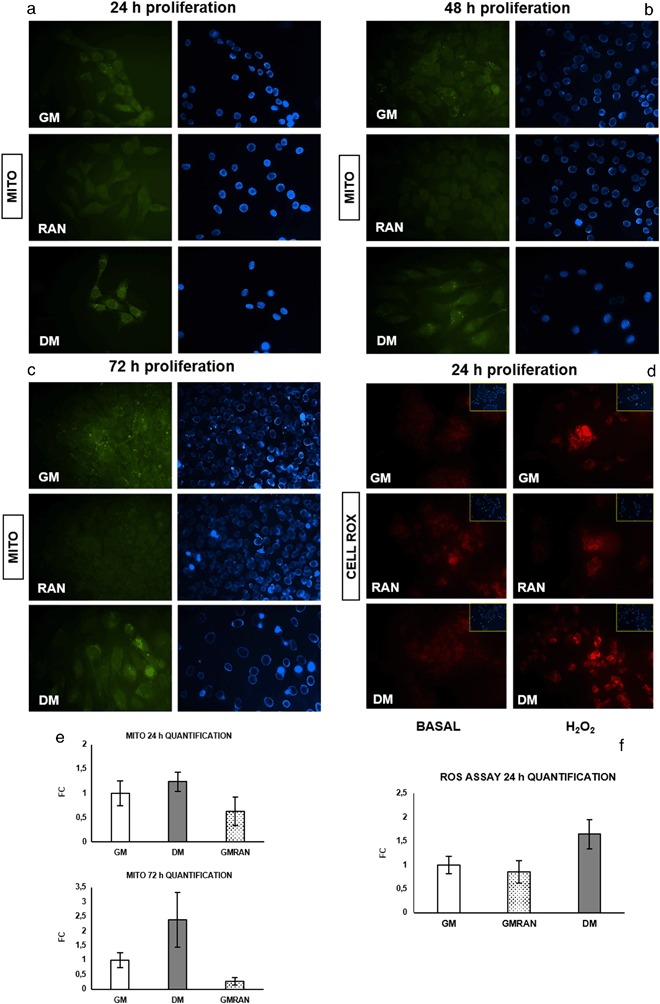
RAN action on mitochondria in C2C12 myoblasts. We analyzed RAN action on mitochondria and ROS levels during the proliferation phase utilizing a specific staining procedure: **a b**, **c** MITO CytoPainter staining (40X): in all proliferative phases (**a** 24 h, **b** 48 h, **c** 72 h), RAN did not modify the number of mitochondria compared with control (GM and DM) as shown in quantification (**e**). **d** Cell Rox^®^ staining (20X): RAN appears to be able to decrease ROS synthesis in the basal state (**f**) and after H_2_O_2_-induced oxidative stress

Figure 4a, b, c shows that RAN did not modify the number of mitochondria compared with control (GM and DM) during all phases of proliferation. As a consequence, since a delicate balance between ROS levels, calcium signaling and antioxidant enzymes expression plays a significant role during differentiation [9], we studied the potential protective activity of RAN in the basal state and after H_2_O_2_ induced oxidative stress. RAN at 24 h of proliferation appears to decrease ROS synthesis with respect to GM and DM demonstrating a potential capacity to induce differentiation (Fig. 4d).

### RAN action during differentiation

**Fig. 5:**
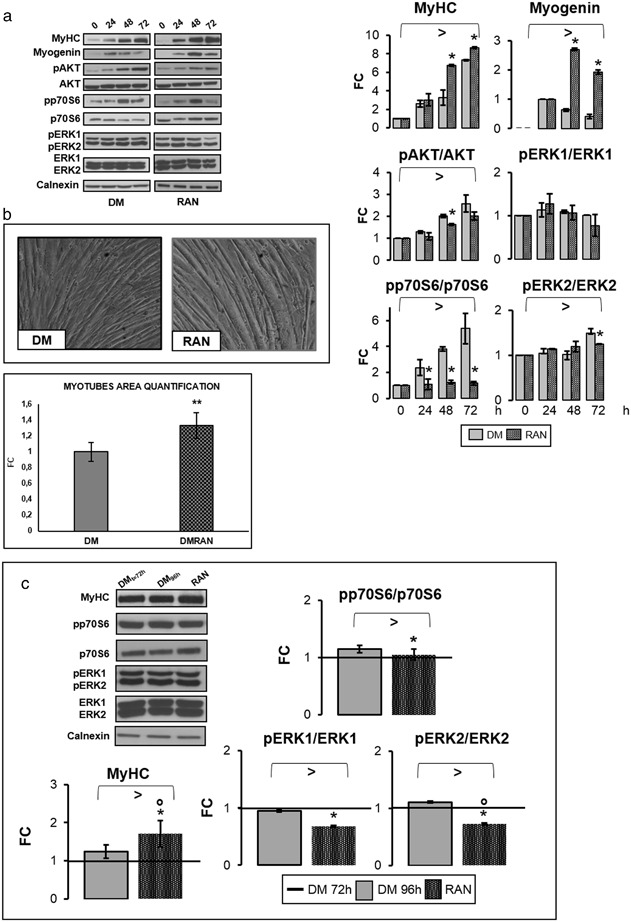
RAN action during differentiation process. To study RAN effects during differentiation, 70 % confluent C2C12 cells were placed in DM with RAN. RAN was not added in the medium (DM) in the control cells. In our in vitro differentiation model, early myotubes appeared after 24–48 h and neo myotubes formation was completed in 72 h. To analyze whether RAN could promote hypertrophy process in neo-formed myotubes, we also treated cells with RAN for 24 h. RAN was not added in the control cells. **a** Western blot quantification data during the differentiation process indicated that RAN significantly improved MyHC and Myogenin protein levels, but did not activate p70S6 and ERK kinases. **b** Phase contrast images at the end of differentiation showed how RAN increased myotubes dimension, as reported in the graph of myotubes area quantification. Objective: 20X. **c** In neo-formed myotubes, RAN raises MyHC protein amount. RAN decreases p70S6 and ERKs kinases activation. Representative immunoblots are shown. Anova test: >*p* ≤ 0.05, *t* test: *p ≤ 0.05 or **0.01 vs. DM or vs. DM_*t* = 72 h_, or °*p* ≤ 0.05 vs. DM_96 h_ (**c**)

The expression of Myogenin and MyHC, late differentiation markers, at 48 and 72 h of differentiation in RAN-treated cells was significantly higher with respect to control (Fig. 5a). Figure 5a depicts the molecular events leading to myogenesis. It is noteworthy that during differentiation RAN does not stimulate AKT and p70S6 expression, appearing significantly lower than in the control. RAN did not modify ERK1 activation and ERK2 phosphorylation in DM was significantly higher only at 72 h (Fig. 5a).

Figure 5b shows a significant increase at the end of differentiation in neo-formed myotubes size (length and diameter) compared with DM control.

In accordance with previous results, we observed a positive effect of RAN on MyHC, without activation of p70S6 and ERKs kinases (Fig. 5c).

**Fig. 6:**
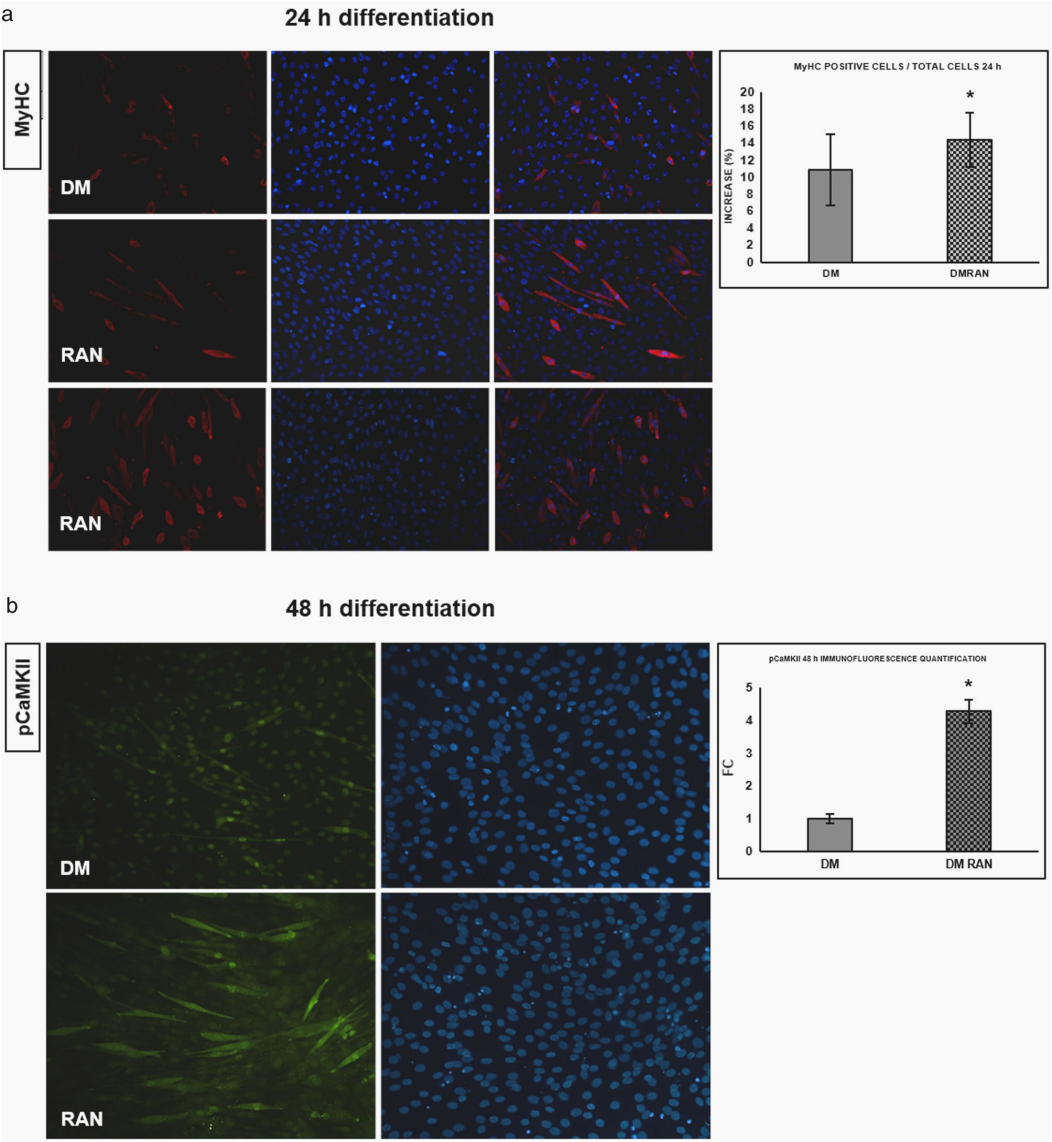
RAN action during the differentiation process: morphological studies. **a** An important marker of muscle differentiation progression is the increase of MyHC positive cells. MyHC immunofluorescence (20X) indicated that in RAN-treated cells the number of MyHC-positive myotubes was significantly higher compared with control (24 h), as reported in the quantification graph. **b** During the proliferation phase we observed that RAN could influence CaMKII signaling. In the same manner, after 48 h of differentiation, RAN could improve pCaMKII protein expression compared with DM control (20X), as reported in the quantification. *t* test: *p ≤ 0.05 vs. DM

Figure 6a indicated that in RAN-treated cells the number of MyHC-positive myotubes was significantly higher compared with control, and confirmed morphological changes mentioned above suggesting an important role of RAN in cytoskeleton reorganization during the differentiation process [20, 23]. It is common knowledge that Myogenin expression requires the activation of CaMKII and Calcineurin: after 48 h of differentiation, RAN improves pCaMKII protein expression compared with DM (Fig. 6b).

### RAN action on mitochondria during C2C12 differentiation

**Fig. 7:**
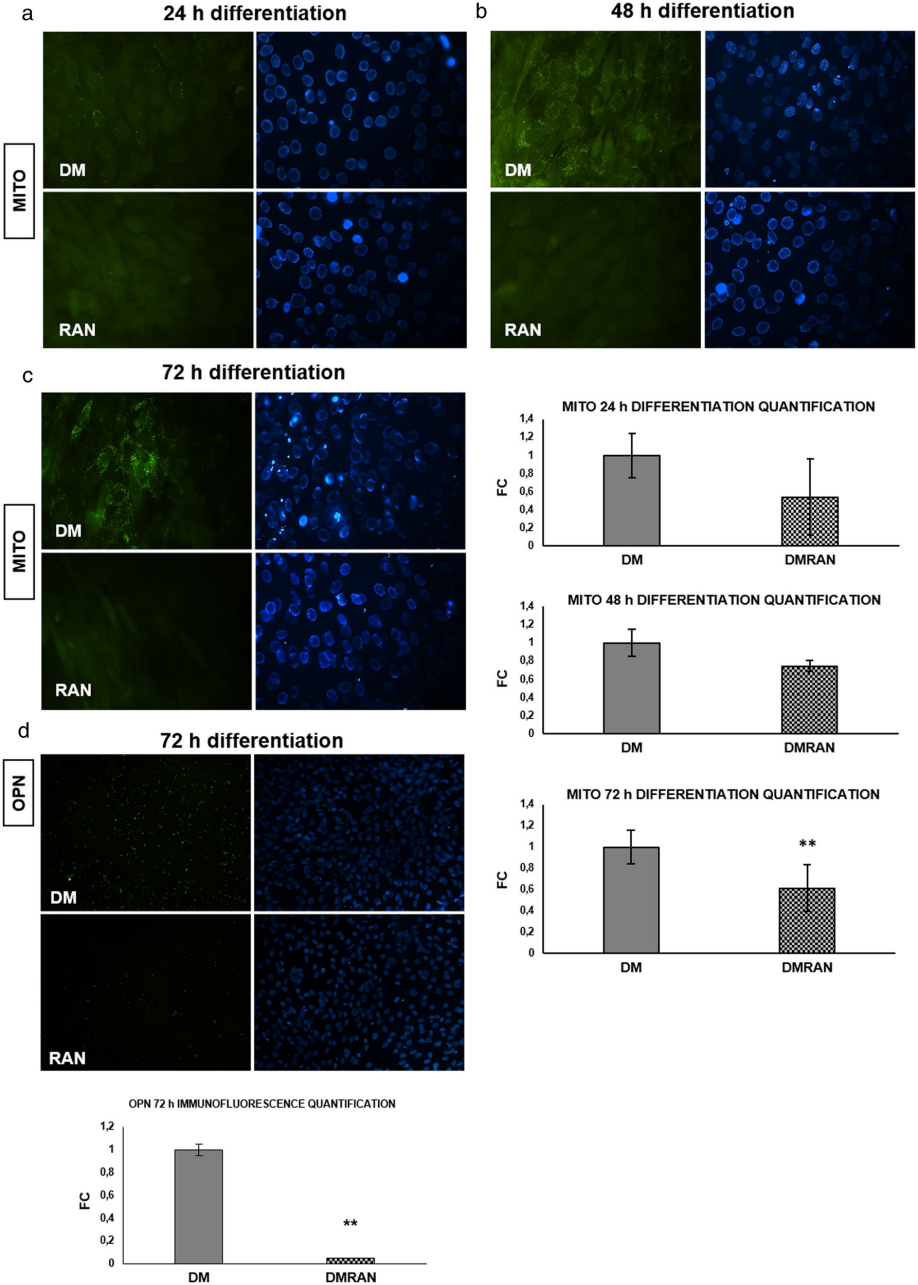
RAN action on mitochondria during differentiation and on OPN protein. We evaluated RAN action on mitochondria during the differentiation phase by specific staining procedure: **a**, **b**, **c** Mito CytoPainter assay and quantification: RAN treatment did not change the number of activated mitochondria [33,34] compared with DM control, in all differentiation phases (**a** 24 h, **b** 48 h, **c** 72 h). Objective: 40X. **d** OPN has been described as a component of the inflammation of skeletal muscle. After 72 h of differentiation RAN treatment significant decreased OPN protein expression compared with DM control (20X), as reported in the quantification graph. *t* test: *p ≤ 0.05 or **0.01 vs. DM

Figure 7a, b, c shows how RAN treatment maintained mitochondrial homeostasis in all differentiation phases.

OPN has been described as a component of the inflammation of skeletal muscle [10]. At 72 h of differentiation (Fig. 7d) RAN treatment significantly decreased OPN protein expression compared with DM control.

## Discussion

The bulk of scientific evidence on the mechanism which may result in RAN to lower blood glucose and function as a novel antidiabetic agent points to the β-cell [1–4]. For the first time, present data suggest an alternative site of action of RAN at the skeletal muscle level. Our initial hypothesis was that RAN could act on the insulin-signaling pathway, as many anti-diabetic drugs do. By contrast, our data show that RAN does not modulate the insulin pathway, but increases the CaMKII phosphorylation, suggesting a role in the activation of the insulin-independent Ca^++^-mediated glucose regulation.

Besides stimulating glucose uptake, at the same time insulin stimulates cell differentiation and hypertrophy through AKT/p70S6 and ERKs pathways. Although AKT/p70S6 and ERKs are not activated by RAN, our data disclose an increase in length and diameter of neo-formed myotubes, which is determined by an augmented expression of late differentiation markers (MyHC and Myogenin), compared with DM control.

Muscle differentiation is crucially regulated by Calcium signals. In fact, the expression of Myogenin requires the activation of CaMKII and the rise of intracellular Ca^++^ levels is a prerequisite for the activation of myocytes fusion process [23]. Seemingly, RAN not only might have a role in the pathogenesis of IR but it might regulate proliferative and differentiation process through CaMKII modulation. Namely, by modulating CaMKII, RAN might control both cellular growth and glucose metabolism.

Furthermore, the increase of intracellular calcium level is responsible for ROS formation through the action of the mitochondrial respiratory chain. During myogenic differentiation, an increment in ROS was constantly described [24]. Mitochondrial ROS formation may have positive and negative effects. In the former case, ROS can activate cellular growth responses and have a role in skeletal muscles during physical exercise, in perceiving fatigue and in physiological aging-induced muscle hypotrophy [24, 25]. In the latter case, excessive oxidative stress may be the cause of several muscular diseases being also one of the pathogenic mechanisms in muscle damage and weakness in the dystrophin deficiency syndrome [8, 26]. In several muscle diseases, the upregulated expression of antioxidant enzymes can be used as a marker of the disease [8].

In skeletal muscle cells, mitochondrial dysfunction causes an increase of oxidative stress eventually leading to IR [27]. Since mitochondria are the principal intracellular source of ROS, our data, revealing RAN ability to reduce the number of mitochondria in proliferation and differentiation phases, demonstrate a promising antioxidant role for this drug.

A few potential mechanisms, alternative to CaMKII activation, by means of which RAN may act as an insulin-sensitizing agent may be taken into consideration. The first one was suggested by the work of Fu et al. [28]. The authors showed that RAN potently increases microvascular perfusion expanding the endothelial surface area available for nutrient and hormone exchanges and significantly facilitating muscle insulin clearance from blood and muscle insulin uptake. The calcium-mediated mechanism and the “microvascular” mechanism may, furthermore, be responsible for determining an improvement of insulin sensitivity.

A second alternative mechanism may rely on OPN production. OPN is a sialoprotein associated with muscle tissue inflammatory, degenerative, regenerative events and IR [29]. Jimenez-Corona et al. proposed OPN as a key molecule associated with, and regulated by oxidative stress, whose overexpression is promoted by ROS [30]. The combination of inflammatory cascades and stress sensitive kinases activation, contributes to impair insulin signaling, demonstrating that oxidative stress has a prominent role in inflammatory response and correlates with mitochondrial damage and IR induction in skeletal muscle cells. Accordingly, it is conceivable that strategies able to decrease mitochondrial dysfunction, oxygen free radicals levels and inflammatory condition may have therapeutic potential. We speculate that, in the presence of an inflammatory process, the effect of RAN on OPN may lead to a diminished inflammation.

Intracellular Na^+^ overload may cause a perturbation of mitochondrial Ca^++^ homeostasis resulting in ROS generation. In fact, when cytosolic Na^+^ concentration rises, mitochondrial Ca^++^ efflux increases through the mitochondrial Na^+^/Ca^++^ exchange, reducing Krebs cycle activity and NADH and NADPH availability [31]. This phenomenon will impair the function of antioxidant enzymes eventually increasing ROS production. A strategy to normalize the elevated ROS levels can indeed be to lower cytosolic Na^+^ concentration via RAN, validating the RAN INaL inhibitory power as a potential antioxidant therapy.

It is evident that Na^+^ concentration and Ca^++^ homeostasis are strongly linked and the subtle balance between the two is responsible for oxidative stress and pathological conditions. The involvement of INaL in maintaining the Na^+^/Ca^++^ balance makes this current a potentially interesting drug target opening the possibility to develop a novel therapeutic option. This is also consistent with data obtained by Burr et al. showing significantly lower fibrosis, fewer infiltrates and lower fiber size irregularity in quadriceps muscles of dystrophic mice treated with RAN [32].

In conclusion, our results indicate that RAN stimulates myogenesis and reduces a pro-oxidant inflammation/oxidative condition, activating the calcium signaling pathway. The main cellular mechanism is the inhibition of Na^+^ current. The newly described mechanisms in the muscle cell may partially explain the glucose lowering effect of the drug beyond a pure β-cell mediated effect.
